# Restoratives and Resuscitants

**Published:** 1894-08

**Authors:** N. S. Hoff


### THE DENTAL REGISTER.

Vol. XLVIII.] AUGUST, 1894. [No. 8.

### COMMUNICATIONS.

### Restoratives and Resuscitants.

BY N. S. HOFF, D.D.S.

Read before the 38th Annual Session of the Michigan Dental Association.

The consideration of the various means and methods of obtaining painless dentistry by this association would not, it seems to me, be complete without some time and thought spent in an especial consideration of the various means and measures that can be called into use, in case the poisonous influences of the anæsthetic make themselves manifest. All persons who have made any considerable use of anæsthetics have discovered that very inconvenient and dangerous symptoms are liable to manifest themselves at times when least expected, and it may be, when the operator is least fortified with antagonizing remedies. Those who have made little or no use of them know from reading of these disagreeable manifestations. Is it not probable that lack of intelligence as to the manner of action and proper use of anæsthetics, also in the selection and administration of restorative remedies, and the management of cases requiring them, deter many from giving their patients the benefit of painless operations even when most urgently indicated ?

In directing your attention to this subject it is not my purpose to attempt an exhaustive treatment of it, but only to bring together in as concise and practical form as I am capable of doing, the approved methods of proceeding in the various accidents occurring from the use of anæsthetics. My personal experience has never brought me in contact with many of the more serious aspects of the subject, and you will, I am sure, pardon my assurance in attempting to write upon the subject at all. But the necessities of the occasion forced the subject upon me, and I have made my best endeavor with the time at my disposal, and the meager material I have been able to get from text-books on the subject.

My endeavor is to present the most widely-accepted theories as to the nature of these difficulties and the best means for meeting them, and where it is at all possible to state some of the principles in definite terms. The treatment of the subject will be confined to the accidents which are liable to occur when using the accepted anæsthetics—chloroform, ether, nitrous oxide and cocaine. For convenience in memorizing we will class the remedial agents under two heads, Restoratives and Resuscitants.

*Restoratives* are the more simple means of stimulation through the reflexes of the vital organs in order to hasten their restoration to normal function, and for those conditions which usually present no alarming indications.

*Resuscitants* are indicated when the conditions require a more or less prompt and vigorous interference to prevent dangerous tendencies or fatal results. The restoratives are all that are ordinarily called for, yet occasionally more active measures are needed, and when such times come, they come suddenly and with little or no warning. Usually these excessive prostrations are the result of concealed or unknown systemic disorders which make themselves known under the prostrating influences of a general anæsthetic. In order that we may more freely grasp this subject it may be well for us to outline the events which are liable to take place and the means by which they are produced, and under each to outline the treatment and give reason for selecting the remedies indicated.

If we look closely into this subject we will find that it is impracticable to make definite distinctions between the several different effects produced by anæsthetics, for oftentimes two or more of the effects we shall describe will be found in a single case. We also find that the same treatment, that is to say, the same remedies will be indicated in different conditions because of this close association of symptoms and effects produced. The only object in making this distinct classification is that it may help to retain the matter in memory in a systematic and rational form.

The accidents liable to be encountered in the administration of anæsthetics are: syncope, cardiac failure, shock and asphyxia. There are, of course, many other incidental or accidental happenings which have nothing to do with the quality of the anæsthetic or condition of the patient, but which come entirely from carelessness or bungling on the part of the operator or his assistant. These we will not take time to consider as they are such palpable errors of judgment or evidence of incompetency that this paper would not be long enough to enumerate and explain them, and no classification would hold them.

Syncope is the sudden lowering of the blood-pressure to the extent of producing cerebral anæmia and unconsciousness. The blood-pressure may be lowered by the heart itself: (1) by the lessened force and frequency of its beat; (2) by the action of the depressor nerve on the vaso-constrictor center, inhibiting its action to make it accord with a feeble or depressed heart; (3) by emotional excitement or shock which cause inhibition of the vaso-constrictor center; (4) by the action of an anæsthetic on the heart producing a direct depressing effect upon the heart muscle or ganglia, and through its afferent depressor nerve an unusual effect is produced on the already excited vaso-motor center.

In syncope the manifest symptoms are sudden blanching of the skin, relaxation of the muscular system, apparent unconsciousness and feeble pulse. The respiration is continuous but shallow. The patient has neither power nor inclination to stand or sit upright, but falls limp and relaxed. These symptoms are due to the depleted or clogged condition of the blood-supply in the central nervous system. Usually the blood collects because of gravitation in the abdominal vascular system which is largely relaxed and dilated. Sometimes there is lack of tone in the arterial system and consequent stasis in the arterioles and capillaries.

Evidently, then, the remedies indicated are such as will restore the relaxed arterial and cardiac systems, and supply the brain with proper nutrient blood. The force of gravity has much to do with the depleting of the brain, especially if the patient be standing or sitting upright. When the extensive vascular system of the abdomen is relaxed, it is capable of containing a large part of the entire blood-supply of the system ; and in syncope the blood quickly finds its way to this part of the body causing a depletion of the more elevated portions of the body. In order that this condition may be corrected the patient must be placed in a recumbent position, and if possible with the head a little lower than the body, but in an easy and natural position. In addition, such remedies should be administered as will increase the power of the heart-beat. Oftentimes simple reflex stimulation is all that will be needed. Draughts of cold air, washing the face with a towel or sponge wet with cold water, the inhalation of volatile irritants such as spirits of camphor or aqua ammonia. If the syncope is more profound and does not yield readily to these remedies, inversion, sometimes with a pendulum movement, or nitrite of amyl will usually give quick response. This is a poisonous drug and should be used with discretion. It is ordinarily administered in from three to five drops, by placing it on a napkin or handkerchief and allowing the patient to inhale three or four times the vapor from the napkin held a few inches from the nostrils. If used in this way and then withdrawn there is a decided quickening of the heart-beat and of respiration. But if its use be continued its action would be harmful, as it produces further dilation of the arterial system. Its use would be indicated in syncope from shock in patients of average good health where the arterial tone was normally good. In chronic invalids suffering from diseases which have impaired the circulatory and nervous systems, and where the patient does not quickly respond to reflex restoratives, and there seems to be no continuous improvement under the most active reflex stimulant, a restorative having a more positive and continuous action is indicated. Such a remedy we have in tincture of digitalis which is a powerful heart stimulant and also excites the vaso-constrictor nervous system. The action of digitalis is to render the heart-beat somewhat slower but of very much more force, at the same time its action upon the vaso-constrictors contracts the arteries of the abdomen so that they drive the venous blood back to the heart and lungs, and by raising the blood-pressure throughout the entire system the normal blood circulation is restored, and consequently its function. Digitalis, either by direct action or because of its action upon the circulation, causes more or less quickening of respiration. There are two preparations of digitalis which may be used: the alcoholic tincture and the alkaloid digitalin. The tincture is preferable, as digitalin is a more or less uncertain compound, and its use is sometimes attended with considerable local irritation, and the results are not as satisfactory as the tincture. The dose of digitalin is $\frac{1}{50}$ of a grain well diluted. The dose of the tincture is xx to xxx drops injected hypodermically into the arm. When using digitalis the patient should be kept quiet and in a recumbent position, as any effort on his part to rise will bring on syncope, by the effort made in rising and the gravitation of the blood to the abdomen and extremities. The patient should be kept warm, and the beat of heart may be very much increased by the application of cloths wrung out of very hot water and placed on the chest over the heart.

In certain diseases more or less caution should be exercised in the application of restorative remedies, otherwise fatal results may follow. All diseases of the arteries, aneurisms, atheromas and fatty hearts would contra-indicate excessive excitement of the circulation, because of possible hemorrhage, or apoplexia.

Cardiac failure may imply more or less depression of the heart function or entire cessation. It is due to the action of various depressing agents on a normal or good heart, or more frequently to these same depressing influences on a heart weakened by disease or impaired by congenital or accidental defects.

Cardiac failure of a sound heart has been sufficiently considered under syncope. But the action of drugs upon a diseased or deformed heart are sometimes very prompt, and baffle all attempts of restoration or resuscitation. Valvular and some congenital deformities are not liable to lead to cardiac failure even under somewhat prolonged depressing anæsthetics, but such diseases as fatty degeneration, indicated by a feeble and irregular action, lack of power, interrupted breathing, etc., are very unfavorable indications for a depressing anæsthetic, such as chloroform. Cautious practitioners will decline to administer such anæsthetics as are liable to produce excessive heart prostration when the heart is known to be weakened from disease. But it sometimes becomes necessary to make use of these depressing anæsthetics even when the conditions are present; or they may be so obscured as not to be recognized by the operator.

There usually accompanies such conditions more or less disturbance of the circulatory system, and, may be, also, considerable nervous disorder, and the effects are, in some respects, similar to syncope, but more desperate. There generally occurs prompt depression of the circulatory and respiratory functions with their peculiar symptoms, and in fatal cases death occurs very promptly with little or no chance for the application of restoratives, and efforts to revive the patient will be futile. Where there is tendency to impaired circulation from disease the restorative remedy should precede rather than follow the administration, and this practice is specially indicated in the conditions here spoken of. The use of alcohol in the form of whisky or brandy is strongly advocated for this condition. It should always be given before the administration and never afterward. The benefit from it is not so much its stimulant value, but its strengthening or tonic effect enables a weak heart to endure the depressing action of the anæsthetic without succumbing. Some authorities advocate one or two ounces given a short time before administering the anæsthetic, others go so far as to make the patient comfortably drunk.

More definite stimulation and fortification of the vital organs is obtained by preliminary administration of digitalis for a day or two previous to the administration, or hypodermically a few hours previous. If accidents occur after these precautions all that can be done is to withdraw the anæsthetic and apply such restoratives as are indicated. If the heart is affected the stimulants mentioned under syncope; recumbent position, reflex stimulants, hot applications over the heart and digitalis hypodermically. If there be respiratory complications, sulphate of strychnine $\frac{1}{50}$ of a grain in one drachm of water hypodermically. In some cases artificial respiration may be indicated.

The next dangerous condition is known as "shock." This term seems to have a more or less wide application and its seriousness will depend upon its form and extent, and also upon the physical condition of the patient. In many cases there is a condition resembling or tending toward shock, produced upon healthy, young or timid patients by fear. It may terminate in syncope or asphyxia, but usually the application of the anæsthetic produces sufficient excitement and comfortable feeling to ward it off and overcome it entirely. There is a condition known as "traumatic shock," produced by injuries, during which it is very questionable whether an anæsthetic should be employed even to lessen the pain of large operations. Again, there is a condition of shock which occurs during the administration of an anæsthetic, which is produced because of painful impressions made upon the vital nerve centers by the afferent nerves from a tissue upon which the surgeon is attempting an operation without waiting for complete anæsthesia. This occurs because these nerve centers are already much depressed by the anæsthetic, but are still irritable, and when the impression comes it makes so strong an attack upon them that they succumb very quickly. Chloroform and cocaine anæsthesia produce this form of shock oftener than ether or nitrous oxide, because the former irritate and depress the nerve centers, while the latter excite and stimulate them. The symptoms of slight shock are confusion, giddiness, pallor, tumultuous heart-beat, spasmodic breathing, sometimes profuse sweating. Generally these symptoms will pass away without the application of remedial agents. The anæsthetic should be withdrawn, the patient put in a recumbent position and warm wraps applied to the body, clothing should be loosened and mild reflex stimulants applied to the face, fanning and rubbing or stroking the hands, warm applications to the feet, and warm tea if the patient can drink without strangling. In severe shock there is pallor, fainting, coldness, trembling, small fluttering pulse, great mental depression, incoherent speech, cold sweat, nausea, vomiting and often great reduction of temperature.

There is great difference of susceptibility of patients to shock, and of the same person at different times. As a rule, young, full-blooded and active people are exempt from shock under anæsthetics, while people inclined to faint, nervous and anæmic people are more susceptible. Sometimes secondary effects follow severe shock, due to the powerful impression made on the nervous system. They take the form of headache, sleeplessness, disturbed mental function, chorea (irregular convulsion of the limbs), cardiac and vascular disturbances, irregular breathing and general mental and physical depression.

We cannot positively affirm that drugs, such as we use as anæsthetics, produce all the features of surgical shock, but the symptoms above outlined so closely resemble the toxic effect of cocaine that we may be justified in treating these effects on much the same principles as shock from a traumatic injury. Anæsthetics produce inhibition of the vaso-constrictor nerve centers and produce syncope, and large doses of chloroform or cocaine produce paralysis of the central nervous and vaso-motor systems producing severe shock, and often fatal effects.

The treatment indicated for severe shock is the same as above indicated in ordinary shock. In addition stronger reflex stimulation and the use of stimulating enemas, and hypodermic injections to meet the indications; digitalis for failure of the heart and strychnine for respiratory failure. In shock from injury attended with pain $\frac{1}{4}$ grain of sulphate of morphine should be given. This would be indicated in shock from the administration of cocaine. As morphine counteracts the exciting tendencies of cocaine at almost every point. It should be used in all formulæ for hypodermic applications of cocaine as a prevention of the toxic effects of cocaine.

Asphyxia is the most common accident encountered in practical anæsthesia and, in many instances, the most difficult one to manage successfully.

It is usually defined as "suspended animation." This does not convey much idea of the way life is suspended, and we shall need to consider its characteristic action to get a clear conception.

Asphyxia, in general terms, is produced by paralysis of the respiratory nerve center and may occur from doses of toxic drugs, such as hydrocyanic acid or carbolic acid; or from overdoses of various anæsthetics, chloroform, etc.; or from the accumulation of venous blood in the circulatory system. These causes are more specifically stated by Harley, in an orderly system to be:

First: The exclusion of air from the lungs by various obstructions to the air passages, such as pressure on the chest or throat, smothering, paralysis of the respiratory muscles because of injury to the spinal cord, wounds into the chest admitting air, foreign bodies in the fauces or larynx, diseases obstructing the air passages, tumors, œdema of the glottis, accumulated mucus, drowning, etc.

Second: Exclusion of oxygen from the lungs by the inhalation of a harmless gas, such as anæsthetic vapors. In the inhalation of nitrous oxide, all oxygen is excluded from the lungs, and that which the blood contained becomes used up and converted into carbon dioxide which is not destroyed by the nitrous oxide, but circulates as a poisonous element to depress the function of the vital nerve centers.

Third: The inhalation of corrosive or poisonous gases or vapors, such as chlorine and carbonic acid gas.

From this it will be seen that these conditions may be present or result from the use of many of our general anæsthetics. The venous or poisonous blood together with the depressing anæsthetic cause profound impression to be made upon the vital nerve centers. The anæsthetics are present first and produce their characteristic effects first of excitement and then of depression of all the higher nerve centers, according to Jackson, in the order of their organization. The highest, the cerebrum first, then the cord in its sensory function, then the special nerve centers of the medulla oblongata, lastly the motor nerves and muscles. During this regular action of the anæsthetic the blood is continually becoming venous in character for want of oxygen and giving no support to these nerve centers and muscles, and, what is worse, even acting as poisons, causing suddenly the function of the life center to stop and consequently of the vital organs controlled by it. Direct paralysis of the function of respiration and circulation would occur in this way and suspended animation is the result. It is also probable that the heart, arteries, lungs and the other vital organs of the system are greatly influenced by the circulation of this venous blood. The heart muscle and the vaso-motor nervous system would undoubtedly be affected by it and the circulation would relax from muscle and nerve poison producing heart failure and syncope combined; also local effects in addition to the central influence of the anæsthetic and poisonous circulation. We can then state our definition of asphyxia with more clearness, as due to the direct depressing action of a depressing anæsthetic, and of vitiated blood upon the respiratory, cardiac and other vital nerve control centers, and to the local poisonous effects of venous blood circulating in the arterial system, depriving the tissues and vital organs of needed sustenance. Asphyxia may be produced in any one or all of the above ways. The peculiar selective action of different anæsthetics are taken into account when these symptoms appear, and all means adopted for restoration or resuscitation must take into account not only the manifest symptoms, but the peculiarity of the drug's action, and the condition of the patient, as treatment will depend largely upon these considerations. But in asphyxia there is one symptom always prominent and demanding immediate treatment. This is failure of respiration.

There are three methods of resuscitation employed in asphyxia.

First: Remove the anæsthetic and also remove any obstruction to the admission of air freely.

Second: Employ artificial respiration and, possibly, reflex stimulants.

Third: Administer restorative drugs either by mouth if possible, hypodermically, or by enemeta. If there is any apparent or suspected interference with the air passages to the lungs, it should be promptly removed. Foreign bodies may become lodged in the trachea or larynx, such as vomited matter, false teeth, extracted tooth, mouth props, mucus, etc. These are sufficient to continue the asphyxia if not removed. If the muscles of the respiratory apparatus, especially of the glottis, are depressed and there is failure of this function, the head should be thrown back and "with the index finger of each hand upon the corresponding cornua of the hyoid bone, whilst the middle fingers rest upon the angle of the jaw, then press forward and upward, the same force serving to extend the head upon the neck; if this fails to open the glottis, by means of a tenaculum, thrust far back into the base of the tongue, draw it forward." (Wood.) If some foreign substance has become lodged in the air passages and owing to the impossibility of recovering it, as sometimes occurs because of the powerful occlusion of the jaws, the operation of laryngotomy or tracheotomy will have to be performed.

Laryngotomy is a simpler operation than tracheotomy and should be made by the operator where such an operation is demanded to save life and no surgeon is at hand to perform it. This operation is made, according to Errichsen, "by making a vertical incision in the median line between the sterno-hyoid muscles, about an inch in length, exposing the crico-thyroid membrane, which is subcutaneous, then perforating this membrane with a transverse cut, with an ordinary scalpel." This artificial opening should be kept open with a tracheotomy tube. The only danger is from hemorrhage in case the transverse cut is made too long the anterior jugular vein may be severed. Artificial respiration can then be set up until the normal function is restored.

If no impediment to respiration exists in the air passages, reflex stimulants are indicated; the use of volatile irritant vapors, such as ammonia, nitrite of amyl, rubbing the face until warm and then dashing cold water upon it, and if insufficient accompanied with artificial respiration. When artificial respiration is indicated, it will usually suffice to simply raise the patient's arms and then bring them down against his sides, so as to compress the abdominal walls. In raising the arms the air is brought into the lungs and the compression again forces it out. These motions kept up at about the regular rate of breathing will induce natural respiration. In some cases where there is entire collapse a more complete and systematic artificial respiration will need to be used. The Sylvester, Hall or Howard method, one or the other, are generally adopted. These methods are so familiar that no description need be given of them here. In practicing artificial respiration, do nothing roughly or excitedly. The respirations should not be made over fifteen per minute, and compression rather than concussion should be aimed at. The administration of drugs to excite the respiration by affecting the respiratory center is one of the most approved methods of resuscitation. The best drug for this purpose, according to Wood, is strychnine. It should be given in the form of sulphate of strychnine in $\frac{1}{50}$ grain in one drachm of water injected hypodermically. If the respiratory centers are not too deeply paralyzed this will excite them and renew the respiratory function. Digitalis is sometimes used, but it has no specific respiratory stimulating function, and any benefit it may have would result from its action upon the heart. If there be also heart paralysis, both drugs should be given. Although strychnine alone seems from clinical evidence to have a certain amount of value as a heart stimulant.

There are many other remedies advocated as restoratives and resuscitants, and they are used with a measure of success; but our space and time would not permit a discussion of their comparative merits. My effort has been to briefly outline the conditions and their nature so far as this can be made out and to class with each condition the most approved remedies.

If it be considered wrong for one who does not understand the nature of the action of anæsthetics to administer them, it certainly is wrong for any one to administer any anæsthetic without providing adequate restorative remedies, and having them conveniently at hand in case they are required, and for this purpose I would propose the following list of remedies to be kept in a special apartment or cabinet for those who comtemplate the administration of general anæsthetics.

A wide-mouth two-ounce bottle filled one-half with cotton saturated with ammonia; two-ounce bottle of aqua ammonia; two-ounce bottle of aromatic spirits of ammonia; nitrite of amyl in five drop tears; one-half pint of whisky; one ounce tincture of digitalis; sulphate of strychnine in one-fiftieth of a grain tablets; caffeine in three-grain capsules; two, one drachm and in good working order, hypodermic syringes; a small galvanic battery; tongue forceps; gag for opening the mouth; pair of long-handled pharyngeal forceps; tenaculum hook; an instrument for laryngotomy, scalpel, tenaculum hooks and tracheotomy tube.

DISCUSSION.

Dr. A. W. Diack: It is vary rarely that we come in contact with the more serious collapsed conditions, these come into the hands of surgeons in cases where more dangerous operations are performed. Then, up to the present time, we have confined ourselves almost entirely to nitrous oxide, the effects of which are not so serious. But we all realize that with the introduction of cocaine we are more liable to meet with such conditions, especially syncope and heart failure. The paper to which we have just listened is exceedingly valuable if for nothing more than the pathological phenomena so clearly set forth. There are two additional conditions which I would mention.

Cases of epilepsy, just after being operated upon are often thrown into a tetanic state—that is, spasm of all the muscles of the body; this state for the time being is very alarming, as those who have witnessed it are well aware. My treatment would be nitrite of amyl, that being considered a specific.

Sometimes extremely nervous persons will exhibit symptoms so closely resembling cardiac failure that it is only after the usual remedies are applied without effect that we discover the condition to be hysteria instead of shock. My treatment of an ordinary case of shock is to use aqua ammonia. It is harmless, and, in the ordinary cases with which we have to deal, effective. If something stronger is needed I use nitrite of amyl. We are here dealing with a remedy that shoots both ways—especially in cases where patient has weak circulation. This drug has an effect upon the heart in so far as it allows inhibition. It has a depressing effect, however, and is not a stimulant. If this does not have the desired effect, I inject aromatic spirits of ammonia or brandy; brandy is more harmless. Give hypodermically twenty drops with about $\frac{1}{6}$ of a grain of morphine, thus obtaining the combined effects of morphine and brandy. Digitalis I would give very shortly after the brandy. In very serious cases I should call in a physician if for nothing more than to take the responsibility. If a death occurs in our chair we have no right to sign a death certificate and a coroner's inquest must be held—a somewhat embarrassing thing to have happen in one's office. Such a thing will, in public opinion, do much toward lowering the esteem in which we may be held. Therefore, call a physician.

Dr. Hoff has spoken almost entirely of the pathological conditions. I would like to say a word about preventive means. We should look at the causes which bring about those conditions. Persons often faint from fright, mere nervousness. This can be overcome by gentle manipulation; often it is best not to let the patient sit in the chair at all. Bromide of potassium is useful here, also. Again, sheer weakness—from disease or old age—will often produce heart failure. Such cases should be treated with stimulants, or morphine.

When there is traumatic shock—when the nerve or remnants of the pulp have been extracted without an anæsthetic—there seems to be an interval of normal condition a few moments after the operation followed by syncope. During this interval attract the patient's attention—give a glass of water and tell her to take a long drink, get her mind fixed upon something outside of herself. Many time, I have avoided syncope by this method.

In regard to traumatic shock just before the anæsthetic period, I have doubts as to whether there is such a thing.

Those most apt to suffer from syncope are patients whose daily occupation is hard manual labor, where the muscles are exercised all day long. Sudden collapse is more liable to occur in such cases than in any other, unless it be cigarette patients.

Dr. W. T. Williams: I do not think dentists need ever use very powerful anæsthetics. Nitrous oxide and cocaine answer our purpose. However, it is well for the dentist to be qualified to take care of patients should they manifest dangerous symptoms. A novel method employed by a medical professor for restoring a patient after the administration of chloroform struck me as very useful. He placed the patient over his shoulder, back to back, with face down, and with the patient in that position walked quickly about the room. This, he claims, empties the ventricles of the heart, clears the throat and starts the heart to beating.

Dr. Carrow: I do not recall that Dr. Hoff gave in his list of restoratives sulphate of atropin to be given hypodermically in case of extreme shock; if he did not mention it I would like to say that I have been very much gratified with the results of the drug upon the motor system.

The suggestion just offered by Dr. Williams of inverting the patient is a most reliable remedy and one always at hand. I have performed one or two operations upon patients while they were in that position. One was the case of a little child too young to allow me to make use of local anæsthetis. I used chloroform. During the administration of the anæsthetic the child gave every evidence of dying. It was inverted and when it began to breathe again, but before it had come entirely out of the influence of the chloroform, I performed iridectomy. The child was out of the influence of the anæsthetic while I was still performing the operation. Of course, by the use of a tilting chair we can get a similar effect, but it is better to suspend the patient.

Dr. Taft: The question of fortification of the patient before performing operations is one not to be overlooked; indeed, it cannot be emphasized too strongly. Patients should be thoroughly examined; one should become familiar with their idiosyncrasies, should make a study of the constitutional conditions, and such prior treatment given as may fortify against disastrous results.

In connection with the subject of inversion of the patient. I will cite a case described by Dr. Jay, of Richmond, Ind. He had administered chloroform. There was a very sudden sinking away, and it was evident that death was near that patient. He inverted the patient and soon there seemed to be complete recovery. But not long after he noticed a blanching of the face; he immediately resorted to inversion and again carried his burden about the room. This procedure he carried on at intervals for perhaps two hours and a half, and finally succeeded in bringing about complete restoration.

In my own practice during the early days of chloroform I had rather a peculiar experience. A healthy, robust-looking girl came to my office to have some teeth extracted. I called in a physician and she was given chloroform. She seemed to recover all right, but in about fifteen minutes she began to smile, then to laugh, and then to laugh hysterically, and finally she went into convulsions. We succeeded in bringing her out of that condition. But not long after she again began to smile—to me, a most sardonic smile—then came heavy stertorous breathing, and then she fell into a sleep. For three days these various conditions were gone through with. Even on the third day she had two or three slight attacks but after that she seemed entirely recovered. I think there must have been a predisposition to hysterical condition that the chloroform brought out. But that was in the early days of chloroform, when the drug was not so well known as now.

There is one point we should bear in mind. Because we have so many good methods of restoring patients, we should not go on carelessly giving anæsthetics, confident that these methods will answer in every emergency, but in many cases preventive measures should be employed, as I before stated.

Dr. Long, Monroe, Mich.: An ounce of preventive is worth a pound of cure. The operator should be on the alert and as soon as there is the slightest sign of collapse he should administer restoratives, even though the symptoms are not very alarming. This is one of our strongholds, I think.

